# Septic Shock Due to Capnocytophaga canimorsus Infection in a Splenectomized Patient

**DOI:** 10.7759/cureus.13815

**Published:** 2021-03-10

**Authors:** Pedro Oliveira, Maria Figueiredo, Vitória Paes de Faria, Gabriela Abreu, Janine Resende

**Affiliations:** 1 Internal Medicine Department, Centro Hospitalar de Vila Nova de Gaia/Espinho, Vila Nova de Gaia, PRT; 2 Microbiology Department, Centro Hospitalar de Vila Nova de Gaia/Espinho, Vila Nova de Gaia, PRT; 3 Nephrology Department, Centro Hospitalar de Vila Nova de Gaia/Espinho, Vila Nova de Gaia, PRT

**Keywords:** disseminated bacteremia, sepsis treatment, capnocytophaga canimorsus

## Abstract

*Capnocytophaga canimorsus* is a gram-negative rod that is part of the commensal flora of dogs’ mouths. Among splenectomized patients who maintain close contact with dogs, the bacteria can lead to infection and fulminant sepsis even without evidence of a skin breach.

In this report, we describe the case of a 71-year-old woman who had undergone splenectomy 35 years ago. She came to our emergency department complaining of back pain, myalgia, asthenia, and a fever of 40.2ºC. No other symptoms were noted upon her admission. Blood workup revealed hyperlacticaemia, increased C-reactive protein, and lymphopenia. A urinalysis and chest radiography were ordered, with no abnormal findings, and the SARS-CoV-2 test was negative. The patient developed persistent hypotension and drowsiness that did not improve with intravenous fluids. Therefore, she was started on a norepinephrine infusion. Cultures were collected, and intravenous antibiotic therapy was started with amoxicillin/clavulanic acid 2.2 mg and azithromycin 500 mg. Besides all the diagnostic tests, no infectious cause was found. On the second day of hospitalization, she started to deteriorate, and antibiotic therapy was escalated to piperacillin/tazobactam 4.5 g, resulting in a good clinical response. On the third day after admission, thanks to a group discussion, we were able to identify *C. canimorsus *in the patient’s blood cultures. A review of history revealed that the patient was in close contact with her pet dog.

This case highlights the importance of a multidisciplinary discussion, including the microbiology team, in order to reach an uncommon diagnosis. When dealing with splenectomized individuals presenting with the septic shock of unclear origin, a history of close contact with dogs must lead clinicians to consider *C. canimorsus* as a causative agent.

## Introduction

*Capnocytophaga canimorsus* is a gram-negative rod that is present in the mouth of cats and dogs as commensal flora [1,2]. It rarely leads to infection in humans, with an infection rate between 0.5 and 0.67 cases per million [3,4]. This infection mostly affects men over the age of 50, and it is more common among immunocompromised patients, such as patients who have been splenectomized, have functional asplenia, are undergoing corticotherapy, have neoplasms, cirrhosis, or alcohol abuse [1,5,6]. Among infected patients, previous histories of being bitten or scratched by a dog, as well as other types of contact - such as the licking of pre-existing wounds (in up to 27% of cases) - are also common [1]. Infection resulting from exposure to cats is much less frequent [5]. However, the infection has also been reported among patients with no evidence of a skin breach [7]. C*. canimorsus* infection can lead to severe disease, with fulminant sepsis [8].

## Case presentation

In this report, we describe the case of a 71-year-old woman with a history of hypertension and type 2 diabetes mellitus with retinopathy. She had also been splenectomized 35 years ago, after a car accident. The patient presented to the emergency department with complaints of back pain, lower limb myalgia, asthenia, and fever for 24 hours. She had no respiratory, gastrointestinal, or genitourinary symptoms. On examination, she had a fever of 40.2ºC, blood pressure was 157/88 mmHg, and heart rate was 130 bpm. The remainder of her examination was unremarkable. Arterial blood gas analysis revealed hyperlacticaemia (3.1 mmol/L), other values were normal. Laboratory investigations showed increased C-reactive protein (CRP; 15 mg/dL) and lymphopenia (920/µL, 18.3%, but no leukocytosis), acute kidney injury (creatinine 1.04 mg/dL), and increased international normalized ratio (INR; 1.28). Chest X-ray and urinalysis were unremarkable, and a SARS-CoV-2 test was negative.

While still in the emergency department, she developed drowsiness and hypotension (85/50 mmHg). The intravenous fluid was, therefore, administered. Urine and blood cultures were obtained, and empiric antibiotics with intravenous amoxicillin/clavulanic acid 2.2 mg and azithromycin 500 mg were started. The patient was admitted to the intermediate care unit with a diagnosis of septic shock of unknown origin.

She developed persistent hypotension and was started on norepinephrine. Other infectious etiologies (cytomegalovirus, Mycoplasma pneumoniae, Epstein-Barr virus, Chlamydia pneumoniae, and Legionella pneumophila) were excluded. We performed a thoracic-abdomen-pelvic computed tomography that revealed hepatic steatosis and findings of splenectomy. A SARS-CoV-2 test was repeated and confirmed to be negative.

The patient started to deteriorate on the second day, developing type 1 respiratory failure with a need for oxygen therapy, as well as an increased norepinephrine dosage. This symptom was accompanied by a rise in CRP (24.25 mg/dL) and procalcitonin (6.98 ng/mL) levels. Because of her clinical deterioration, antibiotic therapy was escalated to piperacillin/tazobactam 4.5 g every six hours.

Meanwhile, after 11 hours of incubation, two aerobic blood cultures isolated a gram-negative rod. After 24-36 hours, very small colonies grew in a chocolate agar under an enriched 5% CO2 environment. Using a matrix-assisted laser desorption/ionization time-of-flight mass spectrometry (MALDI-TOF) Vitek MS® (bioMérieux, Vila Nova de Gaia, Portugal), *C. canimorsus* was identified. In the next two days, the patient’s condition improved clinically and there was a decrease in inflammatory markers. We repeated blood cultures on the second day of antibiotic treatment with piperacillin/tazobactam, with no growth after five days of incubation. An echocardiogram was performed, which was normal. The patient completed seven days of treatment with piperacillin/tazobactam, and all her dysfunctions resolved. The patient revealed that she was in close contact with her pet dog. We were unable to identify the bacteria’s entry point.

## Discussion

The spleen is a lymphoid organ responsible for filtering microorganisms from circulation [8]. Consequently, splenectomized individuals have a higher risk of fulminant infections even from microorganisms that are normally harmless, such as *C. canimorsus*.

*C. canimorsus* infection can range from self-limiting disease to severe infection and death [8]. It has a wide clinical spectrum that can lead to cellulitis, osteomyelitis, cholecystitis, peritonitis, paravertebral abscess, meningitis, Sweet's syndrome, fulminant bacteremia with septic shock, acute kidney injury, hemorrhagic skin lesions, pneumonia, and endocarditis [1,4,6,9]. The most common manifestation of this infection is sepsis with bacteremia [5], as in this case. Without other manifestations, signs of a dog bite or skin lesions, we did not consider the causative agent for our patient’s septic shock at first. Endocarditis was excluded by echocardiography.

*C. canimorsus* is a slow-growing bacteria; therefore, blood cultures may require an incubation period of up to 14 days (with an average of six days) [10]. This often leads to a delayed microbiological diagnosis [1]. Hence, laboratories must be informed about the clinical suspicion so that the culture medium may be adapted. Additionally, this case highlights the importance of a multidisciplinary discussion, including the microbiology team, in order to reach an uncommon diagnosis. In the absence of other clinical or epidemiological data, a meeting of experts allowed us to reach the correct diagnosis for this patient.

This bacterium is usually sensitive to penicillin, third-generation cephalosporins, carbapenems, clindamycin, doxycycline, and chloramphenicol, and it is resistant to aztreonam, trimethoprim, fosfomycin, and aminoglycosides [1,11]. The production of beta-lactamases has also been described in relation to this infection, so antibiotic susceptibility testing should be performed [12]. We decided to treat our patient with piperacillin/tazobactam because she started to deteriorate. The antibiotic was not stripped because the antibiogram was not readily available.

*C. canimorsus* is one of the most lethal bacteria when it leads to sepsis and bacteremia, with mortality ranging from 25% to 60% and reaching even higher percentages among individuals who present with septic shock [5,8]. This lethality can be explained by the explosive bacterial growth that can be observed just a few days after exposure [5].

Contact with dogs is often described among infected patients - particularly bites or scratches. However, in about 10% of cases, the starting point of *C. canimorsus* infection is unknown [2]. Despite all the efforts we made to collect epidemiological data, other than the close contact with her dog, the mechanism and entry site of the infection remained unknown.

Despite the difficulties imposed by this diagnosis, an increase in the incidence of *C. canimorsus* infections has been observed [5]. Potential explanations for this increase include rising numbers of splenectomized individuals because of motor accidents, increased alcohol consumption, greater numbers of immunocompromised patients, as well as more people with pets that live indoors.

Septic shock can be associated with virtually any microorganism. However, when dealing with splenectomized individuals presenting with septic shock of unclear origin, a history of close contact with dogs must lead clinicians to consider the hypothesis of *C. canimorsus*. The laboratories must be alerted to this possibility. Also essentially, splenectomized patients must be made aware of these risks, have up-to-date with recommended vaccinations, and seek medical attention if they feel sick.

## Conclusions

*C. canimorsus* bacteremia remains rare and difficult to diagnose. It implies a good clinical history and communication between the clinical team and the team responsible for processing microbiological samples. When treating a splenectomized patient with fever who has a pet cat or dog, a possible infection with *C. canimorsus* must be considered - even if potential bite injuries are not visible. Greater attention to this disease in clinical practice remains necessary.
